# Pregnant women with diabetes and their clinician's experience of participating in a pilot randomised controlled trial of corticosteroid administration in late pregnancy: A qualitative study

**DOI:** 10.1111/hex.13930

**Published:** 2023-12-06

**Authors:** Linda Sweet, Vidanka Vasilevski, Lee‐Anne Lynch, Joanne M. Said

**Affiliations:** ^1^ School of Nursing and Midwifery Deakin University Melbourne Victoria Australia; ^2^ Centre for Quality and Patient Safety Research, Institute for Health Transformation Western Health Partnership Melbourne Victoria Australia; ^3^ Maternal Fetal Medicine, Joan Kirner Women's and Children's Hospital Western Health Melbourne Victoria Australia; ^4^ Department of Obstetrics and Gynaecology The University of Melbourne Melbourne Victoria Australia

**Keywords:** acceptability, clinical trial, corticosteroids, endocrinology, feasibility, gestational diabetes, pregnancy

## Abstract

**Background:**

Little research exists to support the administration of corticosteroids to pregnant women with diabetes. Pregnant women are often excluded from clinical trials due to concerns of harm to the foetus.

**Aim:**

This study aimed to understand the experiences of women and clinicians of participating in the **P**revention of neonatal **Re**spiratory distress with antenatal corticosteroids before **E**lective **C**aesarean section in women with **D**iabetes pilot randomised controlled trial to determine the acceptability of the study protocol.

**Methods:**

Women and clinicians participating in the pilot trial were invited to complete a telephone interview regarding their experiences of participating. Qualitative data were collected and subsequently analysed using thematic analysis.

**Results:**

A total of 13 women and nine clinicians were recruited between June 2020 and May 2022 for a telephone interview. Participating in the study was deemed acceptable by women and clinicians. Women chose to participate in the study due to the perceived low risk of harm associated with the intervention and for altruistic reasons. The high level of clinical support and information provided for the duration of the pilot trial was valued by women and clinicians. All clinicians highlighted the importance of conducting the trial to inform evidence‐based practice.

**Conclusions:**

Pregnant women are more likely to participate in clinical trials when perceived risks are low and they are well‐informed during decision‐making. Clinicians will support clinical trials when they perceive a benefit to practice and feel assured that women receive extensive monitoring and support. Incorporating these factors into study protocols is more likely to be successful in recruiting pregnant women and maintaining the engagement of clinical staff for the duration of clinical trials.

**Patient or Public Contributions:**

Patients were invited to be participants in this study. A consumer has been included in the planning and oversite of the large multicentre trial.

## 1 INTRODUCTION

The rates of women affected by gestational diabetes mellitus and diabetes mellitus (type 1 and type 2) in pregnancy are rapidly rising, with over 30,000 pregnancies complicated by diabetes reported each year in Australia.^1^ Birth by caesarean section is also increasing, with one‐third of all babies in Australia born by caesarean section.^1^ Birth by caesarean section is more common in women with diabetes^2^, ^3^ than those without.^4^ Birth by caesarean section is potentially lifesaving, however, it is also an intervention that puts women and their infants at greater risk for morbidity and mortality.^5^ Respiratory morbidity is more common in infants born to pregnant women with diabetes,^4^ or born by caesarean section.^6^ Infants affected by respiratory distress syndrome are more likely to require admission to a neonatal nursery, resulting in maternal separation, low breastfeeding rates, invasive procedures such as assisted ventilation and extended hospitalisation.^7^

Administration of antenatal corticosteroids to women before preterm birth is a valuable and cost‐effective treatment to reduce mortality and morbidity, particularly respiratory distress syndrome in infants.^8^ However, a study of women at risk of preterm birth suggested that exposure to corticosteroids before birth was associated with hypoglycaemia in neonates,^9^ a risk factor for poor neurodevelopment and cognitive outcomes.^10^, ^11^ There is a growing body of evidence suggesting that giving women antenatal corticosteroids before elective caesarean section, both during the late preterm and at term reduces the rates of respiratory distress syndrome in infants.^12^

A gap in the literature is that women with diabetes have been specifically excluded from the majority of studies examining the impact of corticosteroids on infant outcomes.^12^, ^13^ Despite there being no evidence from randomised trials to support the administration of antenatal corticosteroids to women with diabetes beyond 35 weeks, clinicians are starting to use them in Australia and New Zealand.^13^

The ‘**P**revention of neonatal **Re**spiratory distress with antenatal corticosteroids before **E**lective **C**aesarean section in women with **D**iabetes’ (PRECeDe) randomised controlled trial has been developed as an international multicentred randomised, placebo‐controlled trial to assess the role of antenatal corticosteroids before elective caesarean section between 35 + 0 and 38 + 6 weeks in women with gestational or pregestational diabetes. Before moving to this large‐scale multicentre trial, the current substudy was conducted as a component of the single centre pilot trial,^14^ which aimed to determine the acceptability of the trial for both women and clinicians, as well as to ensure that recruitment, data collection and outcome measures for the main trial were robust for up‐scaling.

Little literature exists on pregnant women's willingness to participate in clinical research involving pharmaceutical interventions. This is because pregnant women are often excluded from trials due to ethical issues and fears of adverse events bringing harm to the foetus.^15^, ^16^, ^17^, ^18^ More research on pregnant women is needed to ensure they receive appropriate treatment options that improve health outcomes for themselves and their babies.^19^, ^20^ The available studies suggest that pregnant women are reluctant to participate in clinical trials involving drug interventions,^19^, ^21^, ^22^ but were more likely to do so if there was a perceived benefit to the health of the foetus, or if the drug was already being used in pregnancy.^23^ Antenatal corticosteroids are well known to cause maternal hyperglycaemia in women with diabetes, and hence women with diabetes have usually been excluded from trials investigating the efficacy of corticosteroids.^14^, ^24^

Similarly, little is written on clinician experiences of caring for women enroled in clinical trials investigating the efficacy of medications in pregnancy, however, evidence suggests that staff experience a range of barriers to effectively adhering to study protocols.^25^, ^26^ Not understanding pregnant women's unique needs and concerns regarding clinical research participation, and clinician perceptions of study conduct, may lead to poor trial design and impact recruitment.^20^ As we are conducting, to our knowledge, the first randomised placebo‐controlled trial of corticosteroid administration for pregnant women with diabetes at 35 + 0 and 38 + 6 weeks gestation, a unique opportunity to explore women's and their clinician's perceptions of participating in a randomised trial investigating antenatal corticosteroids in women with diabetes were presented. The aim of this qualitative study was to understand the experiences of pregnant women with diabetes and their clinicians of being involved in the PRECeDe pilot trial.

## 2 METHODS

### 2.1 Design

A qualitative descriptive method was used for this study.^27^ Pregnant women who were participating in the PRECeDe pilot randomised controlled trial and staff involved in the administration of the study were invited to take part in individual semi‐structured interviews.

### 2.2 Setting and sample

The study setting was a large public tertiary health service in metropolitan Melbourne, Australia. The service includes one of the largest maternity hospitals in Australia, now recording over 6500 births per year. It is located in a socioeconomically disadvantaged area and caters to a highly culturally diverse population.^28^

The PRECeDe pilot trial was registered before recruitment (Australian and New Zealand Clinical Trials Registry ACTRN12619001475134). Women were eligible for the pilot trial if they were between 35 + 0 and 38 + 6 weeks pregnant and had either pre‐gestational or gestational diabetes (diagnosed on a 75 g oral glucose tolerance test according to the World Health Organization criteria for gestational diabetes) and were booked for birth by elective caesarean section within 7 days. Women with any contraindications to antenatal corticosteroids or to intramuscular injections, or who had received corticosteroids in the preceding 7 days before randomisation were ineligible. Women who were known to be carrying a foetus with a known major foetal or chromosomal anomaly were also excluded. Women were approached between June 2020 and May 2022 about participation in the pilot trial by experienced clinical research midwives. Women who consented to participate in the pilot trial were then invited to participate in this substudy.

Invitations to participate were extended to 16 clinicians who either provided care for women participating in the trial or were involved in recruiting or discussing aspects of the trial with women. The obstetricians were the first point of contact to introduce eligible women to the study. Endocrinology staff were involved in the clinical management of women during their participation in the trial and were available on call 24 h, 7 days a week to manage any concerns related to glycaemic control for trial participants. Research midwives were involved in recruitment, provision of participant information, consent, provision of research injection packs for administration to women, collection of blood samples and follow‐up questionnaires. The clinical midwife was responsible for administering the injections.

### 2.3 Data collection

Semi‐structured individual phone interviews were used to collect the data. An experienced research midwife, not directly involved in the PRECeDe pilot trial, conducted all the interviews. Interviews with women and staff ranged between 10 and 15 min and were audio recorded. The semi‐structured interview guide is shown in Table 1. All audio recordings were professionally transcribed and checked for accuracy.

**Table 1:** Semi‐structure interview guides for women and clinicians.

Women	Clinicians
How well‐informed did you feel when approached to participate in the trial?	How confident are you in the intent and value of the PRECeDe trial?
What were the reasons you agreed to participate in this trial?	How would you feel about it going to the larger multicentre trial? Why?
Were there aspects of participating that concerned you before agreeing? If yes, what were they?	Do you consider the benefits outweigh the risks for this trial?
Did you discuss trial participation with your family of friends before agreeing? If yes, was this helpful?	Do you feel the women who participated were adequately informed of risks and benefits?
What benefits do you feel you have gained from participating in the trial?	Did you have any problems managing women in the trial given you were blinded from their treatment? If yes, what were they, and how did you deal with this?
Do you feel that overall, it was acceptable to participate in this trial or not? Why?	Do you have any recommendations for the researchers to consider in improving the trial?

Abbreviation: PRECeDe, **P**revention of neonatal **Re**spiratory distress with antenatal corticosteroids before **E**lective Caesarean section in women with **D**iabetes.

### 2.4 Data analysis

Data from women and clinicians were analysed separately. Thematic analysis was undertaken using the steps described by Clarke and Braun^29^ and managed with NVivo® software. The researchers who analysed the data were from nursing/midwifery and psychology backgrounds with expertise in qualitative methods. Quotes are marked with participant type and for the women, whether they received the placebo or the intervention.

### 2.5 Ethical approval

Approval for this study was obtained from the Melbourne Health Human Research Ethics Committee (2019.313) with local governance approval obtained from the Western Health Office for Research. Principles of informed consent, anonymity, confidentiality of information, and the right to withdraw at any time were observed.

## 3 RESULTS

### 3.1 Women

A total of 47 women participated in the PRECeDe pilot trial. Nineteen women provided contact details and consented to a telephone interview after birth regarding their experiences of being involved in the trial. Of these, 13 participated in an interview between May and June 2021. After the pilot trial was complete, the data were unblinded to determine which of the women received the treatment and the placebo. There was an almost even spread of women interviewed between the two arms of the study, with seven having received treatment and six receiving the placebo. There were seven women born in Australia, and six women born overseas, including India (two), Pakistan, Vietnam, Malaysia and New Zealand. The primary themes discussed by the women included in the substudy were communication about the study and seeking advice about participation, and support and monitoring made participation feasible.

#### 3.1.1 Communication about the study and seeking advice about participation

For most women, participating in the trial was their first experience of being involved in a clinical research study. Only one of the 13 women had participated in a clinical study before the current trial.

Most of the women were invited to participate in the study by a treating clinician (e.g., obstetrician, endocrinologist, diabetes nurse).

In one of my appointments, they said that someone might approach you to ask if you wanted to participate in the study, gave me some information to discuss and have a read, and they said that the next time I have my appointment someone would talk to me more in depth about it. (P265, Placebo)

One woman and her partner noticed study advertising materials around the waiting area, which prompted them to ask about the study with their doctor.

There were pamphlets around desks … there was always little packets of jelly beans … that were floating around, so my partner had actually questioned why and what was the purpose behind it, at that stage we were told that there was a study going on, the study was briefly explained and they said if you're interested … so we thought if it's going to be of benefit to anybody who's a diabetic why not? (P001, Treatment)

Most of the women were satisfied with the invitation process and did not report any improvements to be made. Many participants discussed their potential involvement in the study with their family and friends. A few women mentioned the positive influence of family members who were health professionals encouraging their participation. Two of the women made the decision on their own, while others sought further advice from their partner, mother's group, mother‐in‐law, and siblings. For most women, the individuals they discussed participation with were supportive, and they found having these conversations useful; as mentioned here: ‘I think it put like my mind at ease, and also just to get people's input on it and see what they think, but everyone was really positive about it’ (P265, Placebo). Two women indicated that their partners had concerns about participating in the study.

… he didn't really want me doing it, because obviously it's a study … you're still finding answers kind of thing. But he ended up being alright about side effects … he was supportive about it, but he didn't encourage me to do it. (P260, Treatment)

#### 3.1.2 Needing clear information and time to decide whether to participate

Most women felt well‐informed about the study and their participation. Those who had questions reported that they were well answered by the research team: ‘[It was] … very good after I read it [the participant information], I understood a lot, and any questions I had when I spoke to them, they answered them for me’ (P268, Placebo). Women mentioned that they felt respected and did not feel pressured to participate in the trial, ‘even when I didn't want to participate in something … I didn't want to do the part where they put the machines for the sugar readings, and they were fine, I wasn't made to feel bad’ (P259, Placebo). However, two women did not feel well informed as they were invited in late pregnancy and had little time to consider participation.

I would've liked to have been more informed because the information I got, [I] got … pretty much the day of it [receiving the injection]. I wasn't able to sit and have a conversation with someone about the whole thing. (P260, Treatment)

#### 3.1.3 Perceived risks and benefits

Most of the women had no concerns before agreeing to participate in the trial, however, those that did have concerns were worried about the risk to their infants; ‘I was just concerned about my baby, because they told me they're going to give me an injection … like whether it's going to affect the baby or not’ (P266, Treatment). Women asked questions about this and felt reassured by the clarification provided by the research team.

I spoke more to my doctor and then more to the diabetes doctor from the hospital, and then also one of the midwives that were doing the research … And they all kind of said the same thing, so I just came to terms with it. (P207, Treatment)

A few women had minor concerns about having injections. Two women were uneasy about agreeing to participate, which involved having a continuous glucose monitor due to the additional stress of having the procedure, ‘I just didn't really want to get it done on top of everything else. I had a pretty bad pregnancy … so I just … wanted it to be over and done with, all the extra needles and stuff’ (P268, Placebo), or being reminded of glucose readings.

I've been having a lot of problems with my sugar readings towards the end, it was stressing me out, and the idea of having it [the continuous glucose monitor] … I was like, ‘I can't do that because my glucose levels were stressing me out towards the end’. (P259, Placebo)

Women described feeling compelled to participate in the trial for the benefit of future women becoming pregnant as there was little research on the area. As one woman said: ‘They rang me, and I was eligible for it, and I just figured someone's got to do it for research for more women and babies, so if I can do it, I'll do it’ (P267, Treatment). All the women considered participation to cause minimal inconvenience or risk.

I wouldn't say there is a risk, I thought it was quite straightforward, you were told everything that was going to happen, you were told you might get the steroids, or you might get a placebo – I thought it was great, I didn't think there … was a concern. (P259, Placebo)

Most of the women indicated that they had no concerns during their participation in the trial. One woman experienced side effects following the injection, ‘I had lumps, my arms went really hot, like sunburnt looking, they were hot for ages after actually, even after an ice pack. And then I bled [at the injection site] a fair bit’ (P260, Treatment). Another woman (P051, Treatment) experienced a spike in blood sugars in the evening following the first injection, but these resolved. One woman was expecting the continuous glucose monitor to be obtrusive but was pleasantly surprised when it was applied.

When they said I had to be monitored … I thought I'd have to carry a monitor on me, but when they showed me, it was just a little thing in my arm and it didn't hurt at all, yeah, I felt at ease. (P265, Placebo)

Almost all the women identified that they experienced no personal benefit from participating in the trial, however, perceived supporting research and helping future pregnant women were benefits.

I still don't know what I was injected with, so to me there's no benefit whatsoever … In the long run, once they've got their study finalised, I would say once we know whether it's a positive or a negative to do the procedures that were done, then yeah it would be a benefit. (P001, Treatment)

One woman noted that the information provided during the study and having access to an endocrinologist directly benefited her.

Before I had the diabetes, I didn't know anything about it. And what else put my mind at ease was they assured me that if anything were to go wrong or any discomfort or if I felt ill, there was always someone that I could call 24/7. (P265, Placebo)

#### 3.1.4 Support and monitoring made participation feasible

There was high acceptability of the study, and the reasons for this were that questions were readily answered, clinicians did not dismiss any concerns, and the process for participating was straightforward, as one participant described, ‘they were always happy to answer questions … they didn't ignore anything … it was an easy process, just filling in paperwork and having whichever injection … so it was easy’ (P001, Treatment). The most identified reason for the acceptability of participation in the study was that the procedures resulted in no harm. Several women mentioned that the lack of research in the area also made the study acceptable. ‘Because it's just helping research … it wasn't harming anyone like it wasn't harming me or the baby’ (P208, Placebo). Some women indicated that they would participate if they were to become pregnant again, however, one woman was uncertain, as she experienced raised blood sugars and did not feel well after the first injection.

### 3.2 Staff

Of the 16 staff invited, nine participated in an interview, including two obstetricians, three endocrinologists, three research midwives and one clinical midwife. The primary themes discussed by staff were their confidence in the trial, recruiting women, managing the care of women in the trial, and the prospect of moving to a large multicentre trial.

#### 3.2.1 Confidence in the trial

All staff agreed that the trial was answering an important research question that would add value to clinical practice. Participants recognised the benefit of corticosteroids for preterm births but indicated the need to determine their efficacy for neonates of women with diabetes who are having a planned elective caesarean beyond 35 weeks gestation. It was believed that the PRECeDe trial would help fill this gap.

I find it's a valuable study … and it's interesting to see that with all the research, we know that steroids are commonly used under 35 weeks, and between the 35 to 39 weeks gestation it's sort of hit and miss who recommends it and who doesn't. So, it's sort of a bit of a grey area that I think that the study would be really good to see whether it's actually beneficial. (S04, Research Midwife)

Staff members reported several potential risks that may impact women in the study. One clinician was concerned about giving steroids to women beyond 38 weeks gestation as it was not included in hospital guidelines, ‘there's the potential of causing harm by giving them the steroids, when actually the hospital policy says we shouldn't be giving them, or hospital policy says they don't need it’ (S01, Obstetrician).

The impact of steroids on diabetes control in women and raised blood sugars requiring infants to be admitted to the special care nursery were also identified risks. Conversely, the risk of not giving steroids when they may reduce the need for an infant to require respiratory support was noted.

… the risks for women and the babies, it comes down to … whether or not corticosteroids have an adverse effect on their glycaemic control, and I suppose the flip side whether, if it is the case that corticosteroids do reduce the risks of neonatal respiratory distress, then the risk of not getting the corticosteroids. (S06, Endocrinologist)

Not understanding the long‐term risks of giving neonates steroids was also raised, ‘for the baby there's the risks both of respiratory immaturity if the baby has immature lungs when it's born, given it's being born below 39 weeks, and the theoretical risk at least of the steroids to the young brain’ (S09, Obstetrician).

Staff justified these risks in several ways. They indicated that the prescription of steroids under ordinary circumstances was clinician dependent, so some women would receive steroids and others would not.

… my impression is that there are already some people who would give steroids and some people who would not give steroids – so whether a woman would or would not have got steroids would depend on their randomly drawn clinician of the day. (S05, Endocrinologist)

The close monitoring of women that occurred due to study participation alleviated concerns of staff regarding the woman's wellbeing.

… we follow them up very closely with their blood glucose readings, they've had the endocrinology consultants on call for them 24/7 after they've had their doses, so we've been moderating the risks for the women and for the baby, we keep a very close eye on them, which we do anyway because they're babies of diabetic women. (S02, Research Midwife)

One staff member indicated that there is some challenge in the need to follow strict protocols and balancing that in the context of providing individualised treatment to women. ‘I think that it is a significant probable tension point … an ethical balance between following the protocol which … will help create consistency, but then individualising treatment, and then the tension with whatever [hospital] guidelines pre‐exist (P05, Endocrinologist).

All staff agreed that the benefits of the study outweigh the risks as the findings will inform evidence‐based practice and can validate what clinicians say to women about their care.

I think the benefits outweigh the risks in the sense of moving us significantly towards doing a larger scale trial, which is to answer a really important question … not just about the mothers but about the newborns, in terms of that trade‐off between what we think might be good for the babies, but it may not be … there's actually uncertainty on both aspects … I think the other valuable thing is we're doing this trial in a multicultural multi‐ethnic … population, and you know a significant majority of the data which is published even broadly in this space has not particularly done that. (S05, Endocrinologist)

#### 3.2.2 Recruiting women

The process of recruiting women was relatively easy, especially for women who had received corticosteroids in a prior pregnancy. Women who had not received steroids in the past were found to be more hesitant, asked more questions regarding the study, and took longer to decide.

Most of the women that we approach who have had steroids in a previous pregnancy are very easy to recruit, they're usually pretty keen. Women that … [are having] first babies or not really [had] any experience with something like this, they're a little more hesitant … they need some more time to read the PICF [participant information and consent form] … and ask questions. (S03, Research Midwife)

Staff described how some women wanted to participate provided they received the steroids; however, this was not possible due to the double‐blinded randomised design, and they were made aware of this. Women who were of non‐English speaking background were also difficult to recruit due to lack of interpreters during the COVID‐19 restrictions; before the restrictions, these women were easier to recruit with the support of in‐person interpreters

Through the whole COVID thing it has been a bit difficult to do that, only because the interpreters have been over the phone, and I have found that that's quite challenging just because having a consent form and trying to read off that, which is quite in depth …. (S04, Research Midwife)

Having a clinician invite women helped to gain trust and women and their partners/support persons could discuss their concerns with them before the research team followed them up to obtain consent for participation later in the week.

The clinician will make the initial discussion with them, and we often find that when it comes from a clinician rather than complete stranger, the women are often a little bit happier to listen to the information. And then they let us know if they're comfortable for us to then follow them up with a conversation, either over the phone. Often, they take the PICF and have a chat with their partners, families, and then we will follow them up the next day. (S02, Research Midwife)

The referring clinicians had considerable influence on the number of women who participated, and some clinicians were significantly more likely to recruit women than others.

I think a lot of it depends on the clinician giving them the information. We found that with particular clinicians, we have a really good success rate, with other clinicians, none of the women want to do the study. So, I think it depends on the clinician's attitude towards the study and towards steroids. (S02, Research Midwife)

One staff member indicated that there was too much information for women and was not confident that all of them had read the information thoroughly before consenting to participate.

I don't know whether they actually would understand those risks and benefits as much as I would like them to, because I don't know if women have been given so much [about] long term consequences, things like lung disease or brain development … it's just difficult for them to make sense of it and so I don't know if they truly understand the risks. (S01, Obstetrician)

A midwife who administered the injections felt that women were well informed about the study, she said, ‘they knew what the medication was and what it was for and … chatting with them … they seemed very well informed’ (S08, Clinical Midwife).

#### 3.2.3 Managing women during the trial

A noted challenge was ensuring women received their injections in a timely manner.

The bit that made it difficult was getting staff to actually give the injection in a timely manner. We've had a little bit of reluctance with certain staff … they're busy and we get it, so we try and make it as absolutely as easy as possible, we get everything ready for them, we've got the drug, we've got the needle, we've got the gloves, we've got everything for them. (S02, Research Midwife)

Clinicians indicated that they were concerned about not knowing whether women had received steroids or not, as this would ordinarily inform their management.

… we don't have that ability to act proactively, we have to wait for hyperglycaemia because we don't know if they've received steroid or whether they've received the placebo … there is a challenge in that we have to wait for high sugars to happen. (S07, Endocrinologist)

As a result, another clinician mentioned that they were inclined to be more vigilant of those involved in the trial, as they did not want the women to experience adverse outcomes.

I do think that particularly the first couple [of women] before I'd really reflected and maybe put a bit of a framework around, I did feel, oh not good, they're in the trial, their sugars are high, we need to do something. (S05, Endocrinologist)

Overall, staff felt that the management of the women was not really impacted, as the care they received was the same as would occur in normal practice. Midwifery research staff members identified that their biggest challenge during the study was receiving the postnatal follow‐up information from women, ‘often they just won't answer the phone or reply to emails, but it would be things like questionnaires, mostly just questionnaires postnatally around 6 weeks’ (S03, Research Midwife).

#### 3.2.4 Prospect of moving to a multicentre randomised controlled trial

The staff were enthusiastic about the trial developing into a large multicentre trial and expressed the need for funding to allow this.

… from my perspective it seems very feasible and very worthwhile, because it would answer a very important question that is the role of antenatal corticosteroid for women with diabetes and it's not really something that seems to have been answered before. (S06, Endocrinologist)

They reported that there was a clear need to do a multicentre trial to achieve the study power required to answer the research question. While the importance of expanding the study to other sites was recognised, considerations which may impact on the study findings were also expressed. Specifically, differences in practice and guidelines across study sites were acknowledged which could impact on the success of the study, however, using multiple sites with a clear and agreed protocol would increase diversity in study participants which would enhance study outcomes.

I think we need to expand it, we need to get the numbers so that we can really quantify … to get the rigour for the outcomes … because we have a very specific population here with our pregnant women, so it would be really good to see what the other areas, other demographics, if they're seeing the same thing. (S02, Research Midwife)

Most staff agreed that it was feasible to include Australian and New Zealand sites but were reluctant to suggest other international sites due to differences in definitions of gestational diabetes and clinical management of this group of women.

I think within Australia and New Zealand it could be fairly straightforward because of the homogenous way in which we approach gestational diabetes. But understanding internationally there's different definitions even on what is considered gestational diabetes, and I think that might be a bit more tricky. (S06, Endocrinologist)

Clinical staff mentioned that findings backed by a multicentre trial would make them feel confident about informing women of the treatment and whether they recommend steroids.

Women often take our word for things … as much as we say we shouldn't be biased but actually if you say to women that this is a great thing and it will help your baby's lungs, but don't tell them that it can have effects on baby's brain development then they're not going to know about it, and it's just how we put it depends on the confidence we have in the intervention or the evidence for the intervention – so I would like to have more information to be able to say confidently to women that yes this is a good thing or no this is not a good thing. (S01, Obstetrician)

While not identified as a risk, a midwife administering the injection could potentially identify the solution, ‘the liquid was very different, so I am presuming I know what one’ (S08, Clinical Midwife). She agreed that this could be a risk in expanding the study if the drug vials were not completely blinded.

Staff indicated a few improvements that could be made in the upscaling of the research. These included ensuring clinicians have a better understanding of the research to support recruitment to the study.

It would be great if you know the clinicians had a much better understanding of each of the trials that we do, but that's really difficult to get everyone on top of every single study … maybe … have a blanket information in there, like in their initial antenatal appointment folder … here's a whole bunch of studies you might be potential for … through your pregnancy, with just a little bit of information perhaps'. (S03, Research Midwife)

An endocrinologist indicated that having an electronic form (such as an app) for women to enter blood sugars would be preferable and improve data accuracy.

I mean ultimately it maybe that some sort of electronic form of putting in blood sugars and management might be good. So right now, what happens is the women will call us, we might write down their sugars and what doses we give. (S05, Endocrinologist)

One of the clinicians mentioned that ensuring 24/7 access to an endocrinologist is maintained in expansion of the study was necessary.

I think that there needs to be the willingness and availability of people like myself to be on call, because otherwise … it isn't really sustainable … there needs to be buy in from the endocrinology services at the multiple centres. (S07, Endocrinologist)

Research midwives suggested that if they could administer the injections themselves, it would avoid delays and reduce interruptions to busy clinicians. This could be achieved by blinding the solutions effectively:

It would be good if we … were able to blind it in such a way that we were able to give the medication ourselves… Or have one person in the research team who can be unblinded, maybe, who doesn't participate in the study recruitment, so that they can give the medication. (S02, Research Midwife)

Minimising the amount of postnatal follow‐up or strategies to enhance completion of the postnatal component from women was mentioned repeatedly, ‘If they could get rid of some component of follow‐up [it] would probably be a little bit easier’ (S03, Research Midwife).

## 4 DISCUSSION

There is little evidence to demonstrate whether the administration of corticosteroids before birth reduces the rate of respiratory morbidity in neonates born to women with diabetes. The PRECeDe randomised placebo‐controlled trial was developed to ascertain whether there is a clear benefit, as current practice is often dependent on hospital policy or clinician preferences, and not evidence‐based. This substudy was conducted to determine women's and clinician's experiences of participating in the pilot trial to explore the acceptability of the study protocol.

Evidence suggests that over 50% of women use a drug during pregnancy,^30^ however, only a small proportion of these substances have been adequately researched in pregnant populations. The ongoing exclusion of pregnant women from clinical trials has led to poor access to individualised treatment for women, putting them and their babies at risk of less‐than‐optimal health outcomes.^16^, ^20^, ^23^ Determining pregnant women's motivations and concerns regarding clinical trial participation is thus an important step in designing a successful clinical trial. This is especially poignant considering the recent pandemic. Pregnant populations were at significant risk of harm due to COVID‐19 infection, and rapid evidence to support the safety of vaccination in this group was needed, however, women were hesitant to participate in clinical trials.^31^ Clinicians report challenges in encouraging women to participate in clinical trials and have highlighted several barriers to ensuring a clinical trial is conducted as intended.^22^, ^25^, ^26^, ^32^ Understanding clinician perspectives about their involvement in the administration of clinical research is important in the development and implementation of study protocols.

The findings of this study demonstrated that the trial was highly acceptable to women and clinicians. Women reported their main motivation for participating was altruism. In line with other research, playing a role in improving outcomes for future pregnant women and their babies was a key motivator for participants.^15^, ^18^, ^21^, ^23^ The majority of women perceived no to very little risk of participating in the trial, and this is often a major consideration in women's decision to be involved in a study.^18^, ^21^, ^23^, ^32^ In particular, the knowledge that corticosteroids were already being used in pregnancy was comforting to women, which is consistent with evidence that shows drugs already used in pregnancy are more likely to be acceptable to participants in clinical trials.^23^

Women felt well informed before consenting, however, some identified there was too much written information provided, while others wanted more information regarding the risk of corticosteroid administration in pregnancy. These are important considerations, as findings have suggested that the main motivator for women to participate in clinical trials is feeling well‐informed, and a significant barrier is not clearly understanding risks.^18^, ^33^ Clinicians also had concerns about whether women adequately understood the risks and benefits, identifying an important area for clarification in future recruitment to clinical trials. Most of the women discussed their decision to participate with their partner or other support persons, however, they would have liked these individuals to be involved in the discussion with clinicians and the research team. This pilot trial took place during the pandemic where women could not have their support people attend their antenatal appointments. Partners and support persons of pregnant women can have differing attitudes about trial participation and have a strong influence on women's decisions to participate.^21^, ^31^ Involvement of a woman's support persons in decision‐making discussions may improve the success of recruitment to clinical trials.

The women experienced a few episodes of hyperglycaemia during their participation in the PRECeDe study, and when they did, having access to an endocrinologist at any time during the day or night alleviated their worries. Optional participation in continuous glucose monitoring was not agreed upon by many of the women, as it was perceived as too much of a burden. For women who agreed to continuous glucose monitoring, the application was easy, and with little discomfort. In the future, providing greater assurance to women that the application of continuous glucose monitoring is fast and painless may improve uptake. Overall, the women were very positive about participating in the trial and felt well supported.

Clinicians were also positive about their involvement in the trial. All staff agreed that the study should be expanded to a multicentre trial and that the findings would support evidence‐based practice. The application of clinical research to practice is an important motivator for staff involvement in trial administration.^25^, ^26^ Recruitment of women was relatively easy for the clinicians; however, they did raise concerns about whether the challenge of recruiting women from non‐English speaking backgrounds and ensuring they are sufficiently informed about the trial, such that language does not become a barrier to participation. Having participation materials translated into common languages has been shown to support study recruitment,^33^ and this may alleviate clinician concerns should they not have access to an interpreter during the recruiting conversation.

Staff perceived little risk to women during their participation and agreed the benefits of the trial outweighed the risks. For some clinicians, balancing the need to follow a strict study protocol, while attempting to offer individualised care was a point of tension. Ensuring clinicians are well resourced and supported to effectively manage women's care within the constraints of a study protocol through ongoing education may be of benefit. Maintenance of 24‐h support from an endocrinologist was seen as especially important, this enhanced women's and clinician's sense of ease in proceeding with the trial. It is therefore recommended that similarly designed studies include ongoing endocrinologist support for participants.

Future iterations of the trial may be enhanced by allowing research midwives to administer injections. This would increase efficiency and ensure minimal time and resources are wasted waiting for a woman to be attended to. Postnatal follow‐up of women was an additional strain on staff due to lack of contact, reducing the burden of this component may save resources and time to focus on key aspects of the study.

### 4.1 Strengths and limitations

Desirability bias may have influenced the results, as women and clinicians involved in the study may have been more inclined to provide positive feedback. Including women who declined participation in the trial would have offered a unique perspective; however, this was not possible. The main strength of the study is that it accounts for both women's and clinicians' experiences of participating in the PRECeDe pilot trial which can improve research methodology in the future. The study provides justification for including pregnant women in clinical trials to enhance the very limited evidence available to support the use of drug interventions in this population.

## 5 CONCLUSION

Acceptability to participate in a randomised placebo‐controlled trial of corticosteroid injection before elective caesarean birth among pregnant women with diabetes was high for women and clinicians. The low‐risk nature of the study alongside increased access to information, support and ongoing monitoring for women participating in the trial were key contributors to acceptability of the trial. Future participation in similarly designed studies might be boosted by educating clinicians about the trial and their role, enhancing communication between clinicians and women, responding to women's needs for more information about risks, and including key support persons and family in discussions about participation.

## AUTHOR CONTRIBUTIONS

Linda Sweet, Vidanka Vasilevski, Lee‐Anne Lynch and Joanne M. Said made substantial contributions to the conception and design, or acquisition of data, or analysis and interpretation of data, were involved in drafting the manuscript or revising it critically for important intellectual content, gave final approval of the version to be published. Each author should have participated sufficiently in the work to take public responsibility for appropriate portions of the content. Linda Sweet, Vidanka Vasilevski, Lee‐Anne Lynch and Joanne M. Said agreed to be accountable for all aspects of the work in ensuring that questions related to the accuracy or integrity of any part of the work are appropriately investigated and resolved.

## CONFLICT OF INTEREST STATEMENT

The authors declare no conflict of interest.

## ETHICS STATEMENT

Approval for this study was obtained from the Melbourne Health Human Research Ethics Committee (2019.313) with local governance approval obtained from the Western Health Office for Research. All participants provided written informed consent.

## Data Availability

Research data are not shared. The ethics approval does not allow data sharing. Additional details can be provided on request from the corresponding author.
